# Practice and Attitude of Cigarette Smoking: A Community-Based Study

**DOI:** 10.1371/journal.pone.0092939

**Published:** 2014-04-02

**Authors:** Bahaa-eldin E. Abdel Rahim, Mohamed Salih Mahfouz, Umar Yagoub, Yahya M. H. Solan, Rashad Mohammed Alsanosy

**Affiliations:** 1 Unit of Population Health, Medical Research Center, Jazan University, Jazan, Kingdom of Saudi Arabia; 2 Department of Community Medicine, Faculty of Medicine, Jazan University, Jazan, Kingdom of Saudi Arabia; 3 Department of Primary Healthcare of Jazan, Ministry of Health, Jazan, Kingdom of Saudi Arabia; 4 Substance Abuse Research Center (SARC), Jazan University, Jazan, Kingdom of Saudi Arabia; The University of Auckland, New Zealand

## Abstract

**Background:**

In Saudi Arabia many studies have addressed cigarette smoking from various perspectives. Most of these studies, however, were conducted among males and confined to Riyadh, the capital city. Such limitations have enhanced the need for community-based epidemiological studies that include both genders and various age groups and socio-demographic features, as well as different regions.

**Objective:**

This cross-sectional study aims to assess the prevalence of cigarette smoking and to discuss the association between cigarette smoking habits and socio-demographic factors among community members of the Jazan area in southwest Saudi Arabia.

**Methods:**

A pre-coded questionnaire was designed and tested for data consistency. A well-trained health team was assigned to gather the data from the 30 primary healthcare centers distributed across eight provinces. The response rate was 92.8% (4,326 respondents ≥13 years old). The associations among the subjects' socio-demographic characteristics were examined by the chi-square test. A multiple logistic regression and odds ratios were calculated as well.

**Results:**

A total of 1,017 (23.5%), 1,042 (24.1%), and 3,284 (75.9%) respondents were, respectively, current smokers (TCS), ever-smokers (TES), and non-smokers (TNS). Though current smokers seem to be more prevalent in urban populations (13.8%) than in rural populations (9.7%), the association of urbanization with a current smoking habit is insignificant.

**Conclusion:**

Having fun, relieving stress, and the influence of parents, particularly of mothers, were the main motives that encouraged participants' cigarette-smoking habits. This situation was worsened by the fact that accessing cigarettes was either very easy or easy for over 90% of the respondents.

## Introduction

Cigarette smoking is powerfully addictive and caused over 100 million deaths in the 20^th^ century alone. In the 21^st^ century, if smoking trends persist as expected, one billion people will die from smoking tobacco [1]. The first initiative for tobacco control in Saudi Arabia dates back to the year 1926 when King Abdul Aziz banned tobacco smoking [2]. The Ministry Of Health of Saudi Arabia (MOH) launched the Tobacco Control Program (TCP) on 23-2-1423 H (May 6, 2002), based on Royal Decree No. 8116. The TCP is the cornerstone of the MOH's long-term control strategies against all forms of tobacco use [3]. Such strategies include health education awareness, as well as the establishment of a series of specialized tobacco control clinics across Saudi Arabia. Other tobacco control initiatives include warning labels for each tobacco box, raising the tax to 100%, and banning smoking advertisements in the mass media [3]. Nearly $160 million is spent annually on tobacco purchases in Saudi Arabia [2]. There are numerous studies that have addressed prevalence and the associated health impacts of tobacco use among different Saudi population groups [2], [4]–[7]. Nevertheless, there is only one community-based study that has tackled this issue [8].

**Figure 1:**
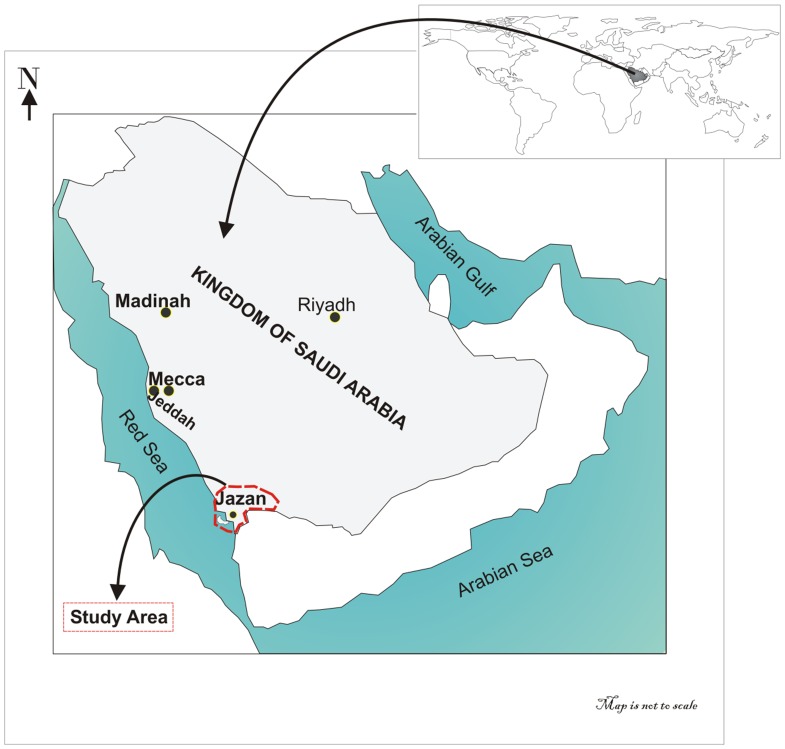
Location of the Study Area.

Due to tobacco's severe effects on human life, tobacco research has been expanded in an attempt to understand the health consequences of cigarette smoking, for both the smoker and nearby nonsmokers [1], [9]–[13]. An independent linkage has been observed between tobacco smoking habits and certain socio-demographic variables [11]–[17]. Some researchers have found that the prevalence rate of substance use among girls increased along with an increasing degree of urbanization, while the prevalence rate of substance use among boys remained constant [15]. In most countries, boys tend to engage more frequently in most adverse health-related behaviors than girls [16]–[18]. However, this pattern does not seem to be universal. It varies with time [19]–[22], the degree of urbanization within a country [23], age, and socioeconomic position [24]. Other researchers demonstrated the mechanism of social and environmental aspects, such as passive smoking, education levels, and smoking habits of partners that control and influence smoking behaviors [25]–[26]. Conversely, with much emphasis placed on establishing data on cigarette smoking prevalence, this study aims to assess the existence of seven independent socio-demographic variables: geographic area (provinces), gender, lifestyle, marital status, level of education, employment, and age group associated with and/or influencing smoking habits among community members in the Jazan area in southwest Saudi Arabia (Figure 1).

## Methods

The Jazan area, with its eight provinces (Jazan, Abu Arish, Fifa, Al-Ardhah, Aldarb, Sabia, Samtah, and Frsan), was chosen as the study area. This area is in the southwestern corner of the Kingdom of Saudi Arabia, with an estimated population of 1,365,110 and a density of 116.97 persons per square kilometer, as per the 2010 census. This study was conducted in accordance to ethical standards. All the participants, including the guardians acting on behalf of the minors/children participants involved in this study, have read, understood, and signed a written consent form. Specifically, this study has been approved by the IRB committee of the Substance Abuse Research Center of Jazan University, Saudi Arabia. The IRB committee includes Dr. Rashad Mohammed Alsanosi and Dr. Mohammed Al-Hassan Taha.

### 2.1 Data

**Figure 2:**
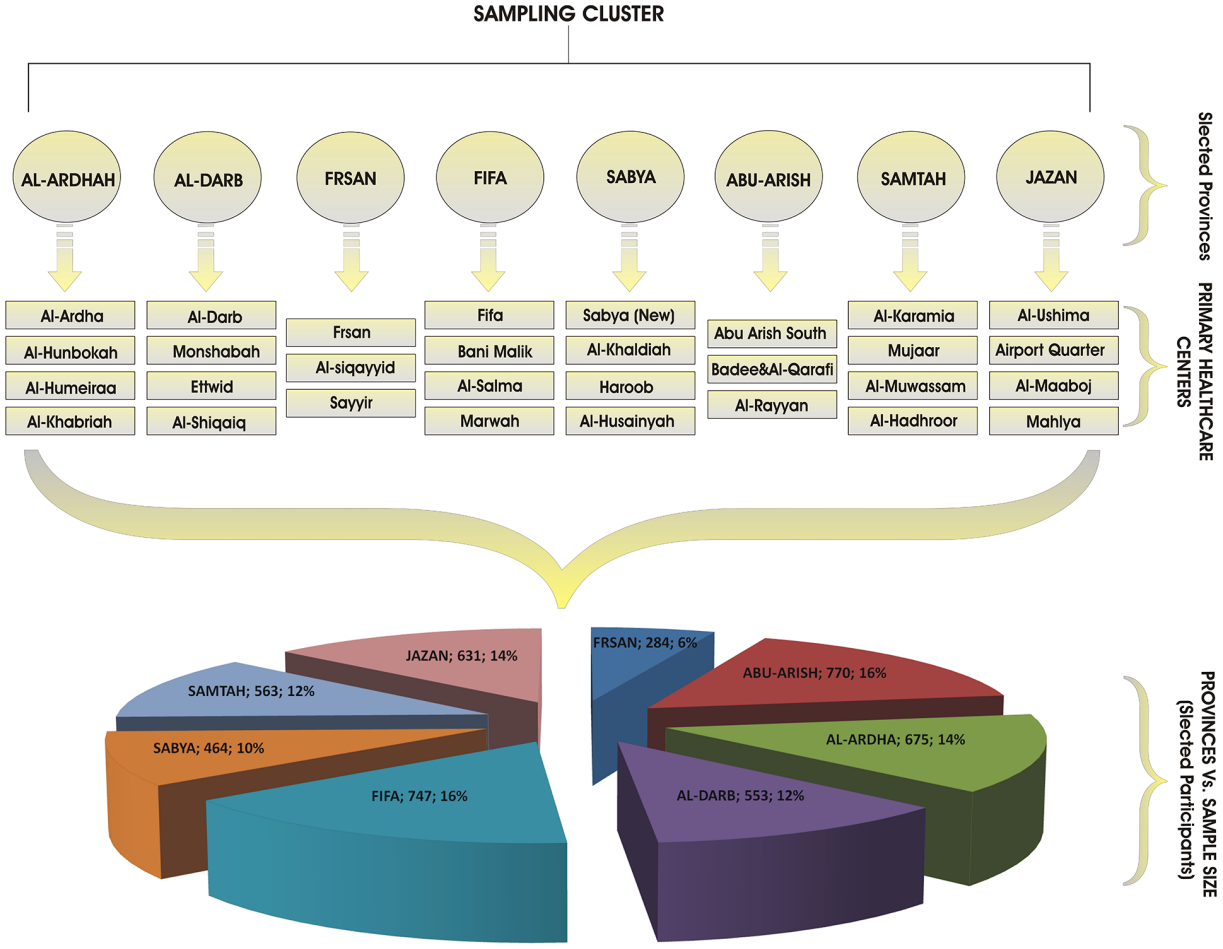
Design of Data Collection Process.

This study is a cross-sectional, health-facility-based survey that was administered in May 2012, using a questionnaire method as the data collection instrument. The sampling was designed to be a three-stage cluster random sampling; each province was considered to be an independent cluster (Figure 2). Stage one was a random selection of eight provinces (Jazan, Abu Arish, Fifa, Al-Ardhah, Aldarb, Sabia, Samtah, and Frsan) out of a total of 14 provinces of the Jazan region. Based on the population density, stage two was a random selection of: (1) four primary healthcare centers within provinces of Jazan, Samtah, Sabya, Fifa, Al-Darb, and Al-Ardhah; and (2) three primary healthcare centers in the Abu Arish and Frsan provinces. Stage three was the selection of study subjects among the attendants of the selected primary health center; this stage was conducted using systematic random sampling. The sample size was 4,660 individuals and determined proportionally to the total population of Jazan region based on the survey of the Central Department of Statistics and Information of Saudi Arabia. All the selected health facilities' attendees (patients and/or co-patients) who were ≥13 years of age were considered to be study subjects. A design effect of 2.0 accounted for the design and respondent-recall bias. A pre-coded questionnaire was designed and tested for data consistency. A well-trained health team was assigned to gather the data. The response rate was 92.8% (4326 respondents).

### 2.2 Variables and Statistical Analysis

The habits of cigarette smoking were categorized as TCS, TES, and TNS. TES were the respondents who had tried even one puff of a cigarette, and TCS were the respondents who smoke cigarettes currently or within a one-month period prior to the date of the interview. In contrast, TNS were the respondents who had never tried smoking in their entire lifetimes.

The statistical analysis was undertaken using Statistical Package for the Social Sciences (SPSS) version 20.0. The prevalence of ever, current, and non-smokers was calculated. The associations between the subjects' socio-demographic characteristics (gender, lifestyle, marital status, level of education, working status, and age groups) were examined using a chi-square test for the categorical data. A multiple logistic regression and odds ratios were adopted to assess the association between current smokers and the socio-demographic indicators; they were computed with 95% CIs. Associations were considered significant at *P*-value ≤0.05.

### 2.3 Ethical Approval

This study was conducted in accordance to ethical standards. All the participants including the guardians on the behalf of the minors/children participants involved in this study, have read, understood and signed a written consent form. Specifically this study has been approved by the IRB committee of the Substance Abuse Research Center of Jazan University, Saudi Arabia. The IRB committee includes Dr. Rashad Mohammed Alsanosi, and Dr. Mohammed Al-Hassan Taha.

## Results

### 3.1 Background Information

A total of 4326 respondents were interviewed across the study area. Abu Arish Province scored the highest percentage of subjects, 753(17.4%), while Frsan was the lowest at 276(6.4%). The respondents were mainly married 2627(60.7%), were males 3069 (70.9%), had stable incomes 2803(64.8%), lived in urban areas 2473(57.2%), lived specifically in the Abu Arish province 753 (17.4%), were aged 19<29 years 1504(25.8%), and had a secondary school education level 1335(30.9%).

### 3.2 Cigarette Smoking Prevalence

**Figure 3:**
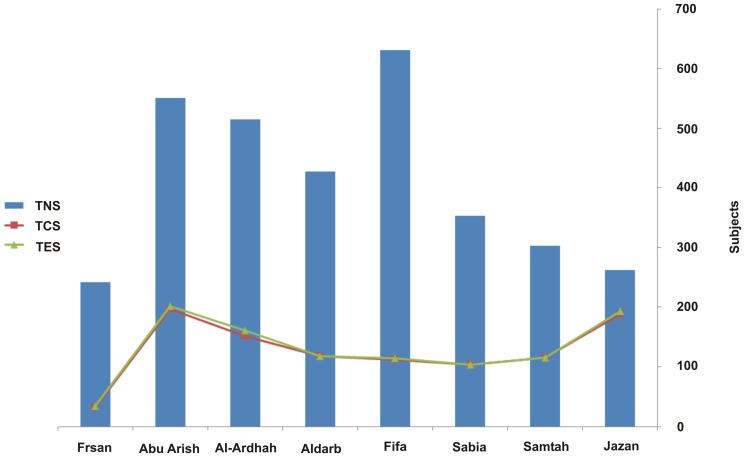
Smoking Status in Eight Selected Provinces within Jazan Area: Total of Non-smokers (TNS) *Vs.* Total of Current Smokers (TCS) and Total of Ever-smokers (TES).

The distribution of all socio-demographic categories and their associated factors of smoking prevalence and prediction is presented in Tables 1 and 2. Respondents (TR) were classified as (1) ever-smokers (TES) if they reported having smoked one or more cigarettes in their lifetimes, (2) current smokers (TCS) if they reported having smoked one or more cigarettes within the 30 days preceding the study, and (3) non-smokers (TNS) if they reported having never smoked in their lifetimes (Figure 3). The associations between dependent and independent variables of smoking habits in the Jazan area are presented in Table 1. A total of 1017 (23.5%), 1042 (24.1%), and 3284 (75.9%) respondents were TCS, TES, and TNS, respectively. Results revealed that the values of TCS and TNS differed significantly at *P<*0.05 across the eight selected provinces (Table 1). The highest TCS, 202(19.4%), was reported in Abu Arish, whereas the lowest, 34(3.3%), was reported in Frsan. TNS scored its highest value of 631(19.2%) in Fifa, followed by Abu Arish, 551(16.8%), while the lowest values were reported in Jazan 262(8%) and again in Frsan 242(7.4%) (Table 1 and Figure 3).

**Table 1:** Significance of Dependent versus Independent Variables of Cigarette Smoking Habits in Jazan Area.

Independent Variables		Dependent Variables									Total	
		TES^1^	TES%	TES out of TR^2^%	TNS^3^	TNS%	TNS out of TR%	TCS^4^	TCS%	TCS out of TR%	TR	TR%
Total		1042	100	24.1	3284	100	75.9	1017	100	23.5	4326	100
Geographic Distribution*	Jazan	194	18.6	4.5	262	8.0	6.1	189	18.6	4.4	456	10.5
	Samtah	116	11.1	2.7	303	9.2	7.0	115	11.3	2.7	419	9.7
	Sabia	103	9.9	2.4	353	10.7	8.2	103	10.1	2.4	456	10.5
	Fifa	114	10.9	2.6	631	19.2	14.6	112	11.0	2.6	745	17.2
	Aldarb	118	11.3	2.7	427	13.0	9.9	118	11.6	2.7	545	12.6
	Alaardhah	161	15.5	3.7	515	15.7	11.9	151	14.8	3.5	676	15.6
	Abu -Arish	202	19.4	4.7	551	16.8	12.7	197	19.4	4.6	753	17.4
	Frsan	34	3.3	0.8	242	7.4	5.6	32	3.1	0.7	276	6.4
Gender*	Males	953	91.5	22	2116	64.4	48.9	935	91.9	21.6	3069	70.9
	Females	89	8.5	2.1	1168	35.6	27.0	82	8.1	1.9	1257	29.1
Lifestyle	Rural	428	41.1	9.9	1425	43.4	32.9	421	41.4	9.7	1853	42.8
	Urban	614	58.9	14.2	1859	56.6	43.0	596	58.6	13.8	2473	57.2
Marital Status*	Single	360	34.6	8.3	1055	32.1	24.4	355	34.9	8.2	1415	32.7
	Married	635	60.9	14.7	1992	60.7	46.0	617	60.7	14.3	2627	60.7
	Divorced	38	3.6	0.9	127	3.9	2.9	36	3.5	0.8	165	3.8
	Widow	9	0.9	0.2	110	3.3	2.5	9	0.9	0.2	119	2.8
Level of Education*	Illiterate	52	5.0	1.2	383	11.7	8.9	49	4.8	1.1	435	10.1
	Read & Write	56	5.4	1.3	177	5.4	4.1	54	5.3	1.2	233	5.4
	Primary	94	9.0	2.2	350	10.7	8.1	90	8.8	2.1	444	10.3
	Intermediate	210	20.2	4.9	569	17.3	13.2	207	20.4	4.8	779	18.0
	Secondary	405	38.9	9.4	930	28.3	21.5	400	39.3	9.2	1335	30.9
	Graduate	209	20.1	4.8	835	25.4	19.3	202	19.9	4.7	1044	24.1
	Postgraduate	16	1.5	0.4	40	1.2	0.9	15	1.5	0.3	56	1.3
Working Status*	Working	753	72.3	17.4	2050	62.4	47.4	734	72.2	17.0	2803	64.8
	Not working	289	27.7	6.7	1234	37.6	28.5	283	27.8	6.5	1523	35.2
Age Group^5^ *	≤19	75	7.2	1.7	299	9.1	6.9	74	7.3	1.7	374	8.6
	20<29	389	37.3	9.0	1115	34.0	25.8	384	37.8	8.9	1504	34.8
	30<39	349	33.5	8.1	985	30.0	22.8	337	33.1	7.8	1334	30.8
	40<49	150	14.4	3.5	520	15.8	12.0	145	14.3	3.4	670	15.5
	50<59	66	6.3	1.5	235	7.2	5.4	66	6.5	1.5	301	7.0
	60<69	12	1.2	0.3	89	2.7	2.1	10	1.0	0.2	101	2.3
	≥69	1	0.1	0.0	41	1.2	0.9	1	0.1	0.0	42	1.0

1 TES: Total of ever smokers (*n* = 1042);

2 TR: Total of respondents (*n* = 4326);

3 TNS: Total of non-smokers (*n* = 3284);

4 TCS: Total of current smokers(*n* = 1017);

5 in years;

*P<0.05.

**Table 2:** Multiple logistic regression model odd Socio-demographic Predictors of Ever and Current Smoking, year 2012.

Independent Variables		Dependent Variables			95% Confidence Interval for Exp(B)		P-Value
			B^a^	Exp(B)^b^	Lower Bound	Upper Bound	
Geographic Distribution	Jazan	Ever smokers	1	1	Reference		
	Samtah		−0.660-	0.52	0.39	0.69	0.0
	Sabia		−0.931-	0.39	0.30	0.53	0.0
	Fifa		−1.411-	0.24	0.19	0.32	0.0
	Aldarb		−0.986-	0.37	0.28	0.49	0.0
	Alaardhah		−0.862-	0.42	0.33	0.55	0.0
	Abu-Arish		−0.703-	0.50	0.39	0.63	0.0
	Frsan		−1.662-	0.19	0.13	0.28	0.0
	Jazan	Current smokers	1	1	Reference		
	Samtah		−0.627-	0.53	0.40	0.71	0.0
	Sabia		−0.886-	0.41	0.31	0.55	0.0
	Fifa		−1.386-	0.25	0.19	0.33	0.0
	Aldarb		−0.941-	0.39	0.30	0.52	0.0
	Alaardhah		−.901-	0.41	0.31	0.53	0.0
	Abu-Arish		−0.692-	0.50	0.39	0.64	0.0
	Frsan		−1.686-	0.19	0.12	0.28	0.0
Gender	Males	Ever smokers	1	1	Reference		
	Females		−1.777-	0	0.14	0.21	0.00
	Males	Current smokers	1	1	Reference		
	Females		−1.837-	0	0.13	0.20	0.00
Lifestyle	Rural	Ever smokers	1	1	Reference		
	Urban		0.10	1.1	0.96	1.27	0.19
	Rural	Current smokers	1	1	Reference		
	Urban		0.08	1.08	0.94	1.25	0.29
Age Group (years)	≤19	Ever smokers	1	1	Reference		
	20<29		0.33	1.38	1.05	1.83	0.02
	30<39		0.31	1.37	1.03	1.81	0.03
	40<49		0.11	1.12	0.82	1.53	0.49
	50<59		0.13	1.14	0.78	1.65	0.51
	60<69		−.812-	0.44	0.22	0.90	0.02
	≥69		−2.317-	0.10	0.01	0.73	0.02
	≤19	Current smokers	1	1	Reference		
	20<29		0.33	1.39	1.05		20<29
	30<39		0.34	1.41	1.06		30<39
	40<49		0.14	1.15	0.84		40<49
	50<59		0.11	1.12	0.77		50<59
	60<69		−.624-	0.54	0.28		60<69
Marital Status	Single	Ever smokers	1	1	Reference		
	Married		−.068-	0.93	0.81	1.09	0.34
	Divorced		−.131-	0.88	0.60	1.28	0.46
	Widow		−1.428-	0.24	0.12	0.48	0.00
	Single	Current smokers	1	1	Reference		
	Married		−.087-	0.92	0.79	1.07	0.26
	Divorced		−.182-	0.83	0.57	1.23	0.36
	Widow		−1.409-	0.24	0.12	0.49	0.00
Level of Education	Illiterate	Ever smokers	1	1	Reference		
	Primary		0.74	2.10	1.49	2.95	0.0
	Intermediate		1.00	2.72	1.95	3.78	0.0
	Secondary		1.17	3.21	2.35	4.38	0.0
	Graduate		0.61	1.84	1.33	2.56	0.0
	Postgraduate		1.08	2.95	1.54	5.63	0.0
	Illiterate	Current smokers	1	1	Reference		
	Primary		0.76	2.13	1.50	3.02	0.00
	Intermediate		1.05	2.85	2.04	3.99	0.00
	Secondary		1.22	3.37	2.45	4.64	0.00
	Graduate		0.64	1.89	1.35	2.64	0.00
	Postgraduate		1.06	2.88	1.49	5.59	0.00

a The coefficient for the constant (the “intercept”) in the null model;

b This is the exponentiation of the B coefficient, which is an odds ratio.

The prevalence of current smoking (PCS) is 935(91.9%) among males and 82(8.1%) among females (Table 1). The majority of TCS were married 617(60.7%) and had a constant income 734(62.2%) (Table 1).

The urban ever-smokers, 428 (9.9%), and rural ever-smokers, 614 (14.2%), differed insignificantly (*P*>0.05), which in turn indicated that urbanization has no influence on smoking initiation. In contrast, the pattern of ever-smoking varied significantly (*P*<0.05) among the categories of each independent variable, including gender [males 953 (22%) and females 89 (2.1%)] and working status [working respondents 753 (17.4%), non-working 289 (6.7%)] as well as province, marital status, level of education, and age groups (Table 1). Similarly, the respondents' lifestyles showed an insignificant influence on the current cigarette smoking habit (*P*>0.05). In contrast, an apparent significant difference (*P*<0.05) was measured for the indicators of the rest of the independent variables (Table 1).

**Figure 4:**
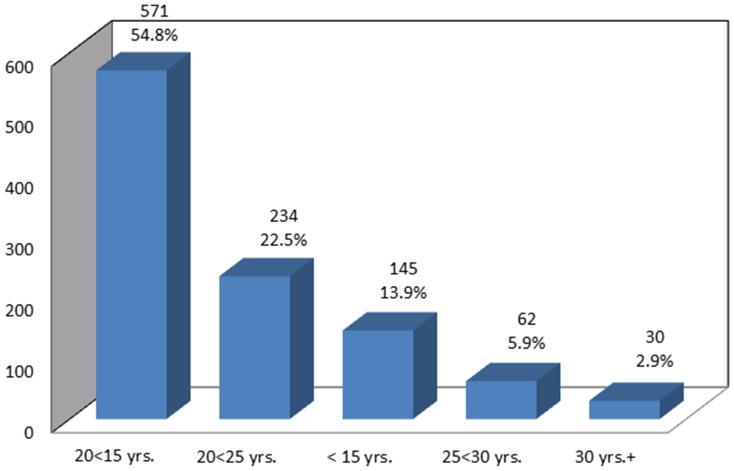
Classification of Smoking Initiation according to the Age Groups.

In response to a question about the age at which the respondents initiated smoking, a total of 571 (54.8%), 234 (22.5%), and 145 (13.9%) respondents reported smoking at the age ranges of 15<20 years, 20<25 years, and >15 years, respectively (Table 1; Figure 4).

### 3.3 Predictors of Current Smoking Habit

#### 3.3.1 Geographic Distribution

Unlike other provinces (*P*<0.05), living in Jazan province shows the highest odds ratio, which in turn can serve as a risk factor for increasing the number of current smokers. However, living in Frsan province shows the lowest odds ratio (OR = 0.19, CI = 0.12, 0.28), which may serve as a protective measure for current smoking habit (Table 2).

#### 3.3.2 Gender

Being female would act as a preventive factor against current smoking habit (OR = 0.159, CI = 0.126, 0.202). In contrast, being male could be predicatively a current smoking addictive factor (Table 2).

#### 3.3.3 Lifestyle

The study investigates whether rural/urban residencies are a formulating risk for being a current smoker. Despite the fact that the prevalence of current smokers seems to be higher in urban populations (13.8%), as compared with rural populations (9.7%), there is no significant association of urbanization with current smoking (Table 1). The multivariate analyses (Table 2) reveal that urbanization acts as a predictive factor for current smoking habit (OR = 1.08, CI = 0.94, 1.25).

#### 3.3.4 Age Group

It seems that exposure to smoking in an adolescent's environment is a significant risk factor (*P*<0.05) for cigarette smoking initiation (Table 1). The age groups 30<39 years (OR = 1.41, CI = 1.06, 1.87) and 20<29 years (OR = 1.39, CI = 1.05, 1.83) show a significant association with current smoking habit (Table 2).

#### 3.3.5 Marital status

A total of 617 (60.7%) respondents were married and current smokers as well (Table 1). Obviously, being married (OR = 0.92, CI = 0.79, 1.07) would increase the chances for the risk of developing a smoking habit (Table 2).

#### 3.3.6 Education

Unlike other levels of education, secondary school students represent 9.2% of the current smokers (Table1). Furthermore, the significantly higher odds ratios (OR = 1.22, CI = 2.45, 4.64) scored for the secondary education, as compared with other levels of education (Table2), indicate that this acts as a smoking accelerating factor.

### 3.4 Motives

There are three main psychosocial motives that encourage smoking habits in the Jazan area (Table 3). These include fun, stress reduction, and imitation of parents. Among TCS, more males smoke just for fun 665(71.1%) and/or tension/stress relief 461(49.3%). Similarly, females—who represent the minority among TCS—smoke for fun, 67 (81.7%), and/or tension/stress relief, 48 (58.5%). On the other hand, nearly one-third of TCS, 368(36.2%), reported that they imitated their parents, especially their mothers {278 current-smokers (27.3%)}.

**Table 3:** Motives Induce Smoking as Reported By Current Smokers (*n* = 1017).

Category	Motives	Males (*n* = 935)	%	Females (*n* = 82)	%	Total	%
Personal	For fun	665	71.1	67	81.7	732	72.0
	Stress Release	461	49.3	48	58.5	509	50.0
Family	Mothers	246	26.3	32	39.0	278	27.3
	Fathers	42	4.5	14	17.1	56	5.5
	Parents	22	2.4	12	14.6	34	3.3
Social	Friends	53	5.7	10	12.2	63	6.2

### 3.5 Regularity of Daily Smoking

A total of 1000 (98.3%) current smokers [919 (90.4%) males and 81 (7.96%) females] reported smoking on a daily basis. Detailed descriptions of the daily smoking habits among the Jazan community are presented in Table 4.

**Table 4:** Regularity of Daily Cigarette Smoking Among Males and Females Current Smokers.

Category	Males	%	Females	%	Total	%
Continuously	557	59.6	23	28.0	580	57.0
2–3 times a day	179	19.1	24	29.3	203	20.0
from time to time	73	7.8	15	18.3	88	8.7
Once a day	58	6.2	7	8.5	65	6.4
1–3 times a week	52	5.6	12	14.6	64	6.3
Non response	16	1.7	1	1.2	17	1.7
Total	935	100.0	82	100.0	1017	100.0

### 3.6 Ease of Cigarette Access

**Figure 5:**
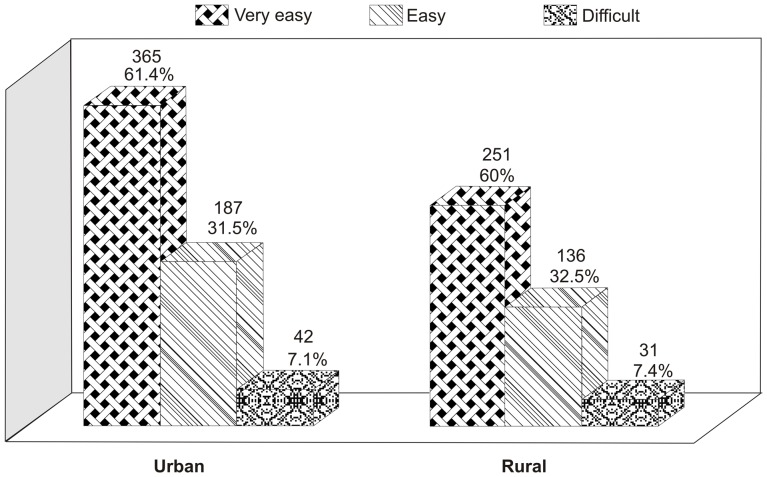
Categorization of Cigarettes Accessibility vs. Lifestyle.

A total of 593 (63.4%) males and 23 (28%) females reported that it is very easy to obtain cigarettes. The results revealed that it is very easy for a total of 251 (60.0%) current smokers who live in rural areas and 365 (61.4%) current smokers who live urban areas to obtain cigarettes (Table 5; Figure 5).

**Table 5:** Cigarette Obtaining and Attitude of Current Smokers.

Category	Very easy	%	Easy	%	Difficult	%	Total	%
Males	593	63.7	289	31.0	49	5.3	931	100
Females	23	28.4	34	42.0	24	29.6	81	100
Total males females	616	60.9	323	31.9	73	7.2	1012	100
Rural	251	60.0	136	32.5	31	7.4	418	100
Urban	365	61.4	187	31.5	42	7.1	594	100
Total Rural & Urban	616	60.9	323	31.9	73	7.2	1012	100

## Discussion

Tobacco in the form of cigarettes is the most prevalent psycho-stimulant substance used worldwide, including in Saudi Arabia, which follows the Framework Convention on Tobacco Control (FCTC). Despite following the FCTC, however, Saudi Arabia has jumped from the 52th rank internationally to become one of the top 10 countries that spend the most money on tobacco products [3], [33]. Unfortunately, this situation seems to have been maintained in Jazan area, where TCS is prevalent among nearly a quarter (23.7%) of the total population, especially those at their productive age. In fact, this percentage is expected to rise, mostly through ease of cigarette access in individual purchasing in retail outlets. In addition, most TCS have a constant income, which in turn can enhance purchase capacity in one of world's highest-income countries according to the International Bank Classification [3]. This situation has been worsened by the average current cigarette selling price in Saudi Arabia (∼$2) being so affordable and far below its counterparts in developed countries [3], [34]. Therefore, tremendous efforts are still needed to reduce tobacco use consistently every year and simultaneously intensify efforts to control the yearly increasing demand for tobacco in Saudi Arabia, including the Jazan region.

In Saudi Arabia, many studies have addressed cigarette smoking from various perspectives. Most of these studies, however, have been conducted among males and confined to Riyadh, the capital city. Such limitations have enhanced the need for community-based epidemiological studies that include both genders and various age groups and socio-demographic features, and different regions [2], [27]–[32]. The overall prevalence of current smoking (PCS) in Saudi Arabia ranges from 2.4% to 52.3% (Median  = 17.5%) across regions. Another previous survey has indicated that total smokers represented 21% of the Saudi male population aged over 15 years; whereas the highest rate of smoking (67%) was reported amongst males aged 21–40 years (34). Strictly, the PCS ranges from 12% to 29.8% (Median  = 16.5%) and 2.4% to 37% (Median  = 13.5%) among Saudi Arabia's secondary-school and university students, respectively [2].

The population of this study included only subjects (patient or co-patient) who were attending various Department of Health facilities, a selection that in turn may have some implications regarding data fullness and completion. In fact, this selection has been made to avoid any social complications that might rise through the extremely conservative nature of the Saudi community, especially in rural and semi-rural areas such as the Jazan region. Even the definition of a rural/urban area used in this study may not perfectly maintain similar and/or homogeneous features within and among provinces. Therefore, this study provides prevalence data on only subjects who attended healthcare facilities. The current study highlights the differences among the statuses of smoking habits (with emphasis on current smokers) relevant to some socio-demographic factors in the Jazan region. Contrary to popular belief [15], [31]–[32], the current study indicates that urbanization has an insignificant influence (*P*>0.05) on current smoking habit, probably because lifestyle differences are slight between rural and urban areas. Conversely, current smoking habit varies significantly (*P*<0.05) by the other independent variables, including geographic distribution, gender, marital status, working status, level of education, and age group.

In consistence with other studies [2], [33], unlike females, males have reported a high rate of daily smoking, which highlights gender as predictively a risk (male) or preventive (female) factor.

Surprisingly, the heaviest smoking attitude (*P*<0.05) was observed among the educated respondents, with 630 of them having at least a secondary education level. In fact, while the PCS among secondary-school students (39.3%) remarkably exceeded the range (12–29%) reported in Saudi Arabia [3], the PCS among the university students (19.9%) remained within the previous range of 2.4–37% [2].

The PCS has noticeably exceeded the rates of previous smoking prevalence investigations [2], [33]. Analyzed by age group, a total of 721 (70.9%) current smokers were young adults aged between 15 and 25 years, which in turn explains the adventurous spirit of a higher proportion of current smokers who smoked just for fun. Accordingly, this factor of being a young male can be a predictive key factor for smoking initiation and/or addiction. This finding is consistent with the previous ranges of PCS in Saudi Arabia [2], [33], but it seems that the smoking habit has become more prevalent amongst young adults (15–25 year) rather than older adults [33]. On the other hand, as expected [2], [33], it seems that males (*P* = 0.00) engaged more frequently in smoking habits than females did, mostly through an adventurous spirit, depressive motives, or imitation of parents. This finding highlights the strong influence of gender as an indicative predictor not only for smoking initiation but also for nicotine dependence; with TCS taken into account, males were remarkably heavier smokers, and somehow this male dominance has been sustained over the years [33]. Among the TCS, males found it very easy 593 (63.7%) or easy 289 (31%) to secure their own cigarette stocks, which was not the case for females. Comparatively, urban areas can act as a predictive indicator for PCS in terms of ease of access.

## Conclusion

Considerable provincial and residential variations were found among the dependent variables. It seems that smoking prevalence was not influenced by urbanization, although there was a slightly higher number of current smokers who resided in the Abu Arish and Jazan provinces, the most urbanized areas in the Jazan District. The highest PCS was noticed among males (935, 21.6%) who were either married (617, 14.3%), working (734, 17.0%), in secondary school (400, 9.2%), somehow living in urban areas (596, 13.8%), specifically from Abu Arish province (197, 4.6%), and in the age group of 19–29 years (384, 8.9%).

In order to design an effective tobacco control policy in any community, it is essential to first understand smoking prevalence and predictors. Therefore, there are obvious associations among the PCS in the Jazan area on one hand, and education levels and working and marital status on the other. These associations highlight the urgent need to investigate the influence of work and family stress on the initiation or addiction of a smoking habit. Furthermore, exposure to smoking in adolescence (i.e. among secondary-school students) is a critical risk factor and an accelerating predictor for smoking initiation. In contrast, being female is a preventive factor for smoking habits. Having fun, relieving stress, and the influence of immediate parents, particularly mothers, were the main motives for cigarette smoking habits. This situation was made worse by the fact that cigarette access was either very easy or easy for a higher proportion of TCS.

## Recommendations

This study recommends, ideally, that a restrictive law enforcement policy be enacted and implemented against tobacco smoking. Since affordability is a crucial factor for PCS, the government should consider doubling or even tripling the current cigarette selling price. Furthermore, it should design and implement a community-based health education program that targets family members, especially youths and mothers. At the secondary-school level, a school-based intervention should be introduced to widen students' scope of knowledge about cigarette smoking's hazards and adverse effects on personal and public health. In addition, anti-tobacco educational efforts and messages should be included in the school curriculum.
