# Correction: Practice and Attitude of Cigarette Smoking: A Community-Based Study

**DOI:** 10.1371/journal.pone.0105273

**Published:** 2014-08-01

**Authors:** The PLOS ONE Staff

There is an error in the first sentence of the Methods section. The correct sentence should read: The Jazan area, with its eight selected provinces (Jazan, Abu Arish, Fifa, Al-Ardhah, Aldarb, Sabia, Samtah, and Frsan), was chosen as the study area.

There is an error in the penultimate sentence of section 2.2 Variables and Statistical Analysis. The correct sentence should read: A multiple logistic regression and odds ratios were calculated to assess the association between current smokers and the socio-demographic indicators; they were computed with 95% CIs.

There are errors in Figures 2, 4 and 5. In Figure 2 there is a spelling error in the Provinces Vs. Sample Size title; it should read “Selected Participants”. In Figure 4, the axes labels are missing. In Figure 5, the values for rural lifestyle are incorrect; the correct values should read 60.04%, 32.54% and 7.42% for very easy, easy and difficult, respectively. Please see the correct Figures 2, 4 and 5 here.

**Figure 2:**
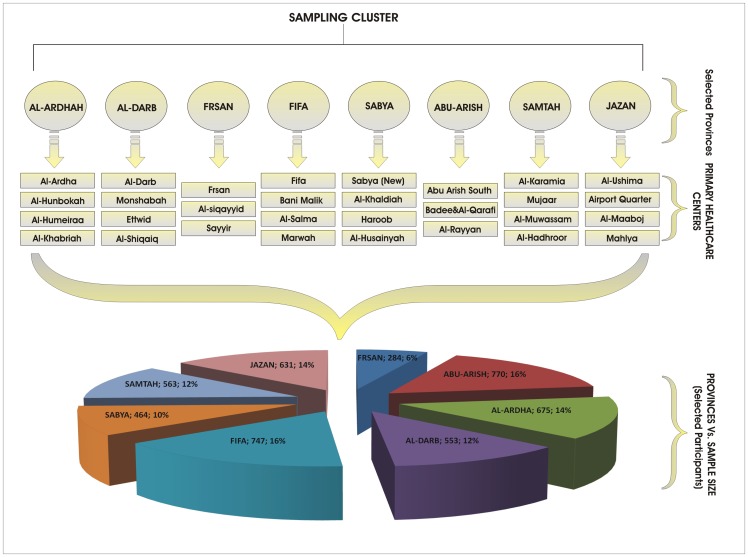
Design of Data Collection Process.

**Figure 4:**
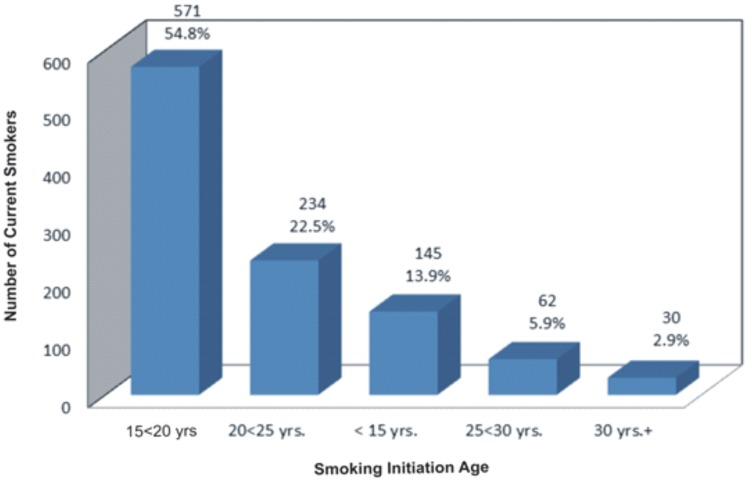
Classification of Smoking Initiation according to the Age Groups.

**Figure 5:**
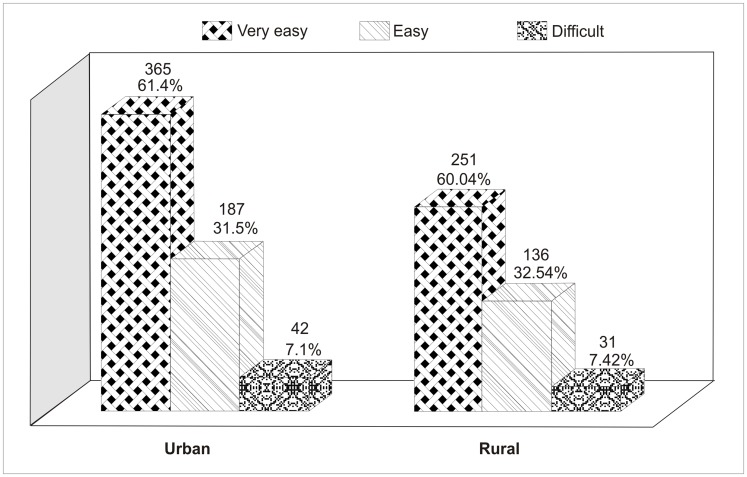
Categorization of Cigarettes Accessibility vs. Lifestyle.
